# Healthcare-associated infections and antimicrobial use in a Greek tertiary hospital: two cross-sectional point-prevalence surveys in 2012 and 2022

**DOI:** 10.1017/ash.2026.10912

**Published:** 2026-10-08

**Authors:** Nikoletta Th Karavasili, Sofia Dimou, Alexandros Argyriadis, Eleni N. Albani, Maria Saridi, Stavroula Tsiara, Georgios Manomenidis

**Affiliations:** 1 Nursing Department, Democritus University of Thrace: Demokriteio Panepistemio Thrakes, Alexandroupolis, Greece; 2 University of Patras - Patras Campus Rion: Panepistemio Patron, Greece; 3 Nursing Department, Hellenic Mediterranean University: Elleniko Mesogeiako Panepistemio, Greece; 4 Nursing Department, University of Patras - Patras Campus Rion: Panepistemio Patron, Greece; 5 University of Thessaly - Larisa: Panepistemio Thessalias - Larisa, Greece; 6 University of Ioannina: Panepistemio Ioanninon, Greece

## Abstract

**Objective:**

To describe and cautiously compare two cross-sectional snapshots of healthcare-associated infections (HAIs) and antimicrobial use (AU) at the same Greek tertiary hospital in 2012 and 2022.

**Design:**

Secondary analysis of two cross-sectional point-prevalence surveys using European Centre for Disease Prevention and Control protocol versions 4.3 and 6.1.

**Setting:**

A public tertiary-care university referral hospital in northwestern Greece.

**Patients:**

Eligible inpatients present in participating wards before 08:00; 276 patients were included in 2012 and 391 in 2022.

**Methods:**

Patient-level HAI prevalence was the primary comparison, and AU prevalence was secondary. Infection site, microbiological, susceptibility, antimicrobial indication, and prophylaxis findings were analyzed descriptively because only aggregate outputs were available.

**Results:**

HAI prevalence was 23/276 (8.3%) in 2012 and 48/391 (12.3%) in 2022 (prevalence ratio [PR], 1.47; 95% confidence interval [CI], 0.92–2.36; *P* = .126). AU prevalence was 147/276 (53.3%) and 206/391 (52.7%), respectively (PR, 0.99; 95% CI, 0.86–1.14; *P* = .937). Pneumonia was the most frequently recorded infection-site category in both surveys. Surgical prophylaxis exceeded 1 day in 51/58 (87.9%) courses in 2012 and 47/48 (97.9%) in 2022. Protocol-year carbapenem non-susceptibility markers were frequent among small numbers of tested isolates.

**Conclusions:**

Both snapshots showed substantial HAI and AU burdens. The observed HAI difference cannot be separated from changes in patient mix, season, the COVID-19 pandemic, surveillance practices, and protocol definitions. Prolonged surgical prophylaxis was highly prevalent at both survey dates and represents a clear stewardship target.

## Introduction

Healthcare-associated infections (HAIs) cause substantial preventable morbidity, mortality, and disability and remain a major patient-safety burden in acute-care hospitals.^1,2^ Antimicrobial resistance further complicates HAI treatment, particularly in tertiary and intensive-care settings where severe illness, invasive devices, and broad-spectrum antimicrobial exposure converge.^3^ Infection prevention and control (IPC) and antimicrobial stewardship (AMS) are therefore complementary strategies: IPC reduces acquisition and transmission, whereas AMS promotes appropriate agent selection, dosing, route, and duration.^4^

The ECDC point-prevalence survey (PPS) program provides standardized estimates of HAI and AU prevalence and describes infection sites, microorganisms, non-susceptibility markers, prescribing indications, and prophylaxis practices.^2^ In the 2022 Greek national PPS, 1,175/9,707 hospitalized patients (12.1%) had at least 1 HAI and 5,376/9,707 (55.4%) received at least 1 antimicrobial. HAI prevalence varied from 14.3% in tertiary hospitals to 7.5% in secondary hospitals and 3.5% in pediatric hospitals; corresponding AU prevalences were 64.9%, 53.5%, and 39.5%.^3^ These differences underscore the importance of hospital type and case mix when interpreting institution-level PPS findings. Point-prevalence surveys have demonstrated their value for identifying infection-prevention and antimicrobial-stewardship priorities across European hospitals and regions.^5–8^

The value of the present analysis lies in identifying institution-specific priorities rather than estimating a national trend. We therefore described and cautiously compared two cross-sectional snapshots of overall HAI and AU prevalence obtained at the University Hospital of Ioannina in 2012 and 2022. Specialty patterns, infection sites, microbiological findings, protocol-specific non-susceptibility markers, antimicrobial indications, and surgical-prophylaxis duration were examined as secondary descriptive outcomes.

## Methods

### Study design and setting

This secondary analysis used aggregate outputs from two hospital-level cross-sectional PPSs conducted at the University Hospital of Ioannina, a public tertiary-care university referral hospital serving northwestern Greece. The 2012 survey was conducted from June 5 through July 5, 2012, as part of the first European PPS and followed ECDC protocol version 4.3.^9^ The 2022 survey was conducted from November 7 through 21, 2022, as part of the third European PPS and followed protocol version 6.1.^10^ Each participating eligible ward was surveyed once during its respective survey period. The study was not designed as a before-and-after evaluation, and no intervention or policy change was defined as the exposure.

### Population and data collection

All inpatients admitted before 8:00 a.m. and present when the ward was surveyed were eligible. Patients admitted after 8:00 a.m. and those treated exclusively in emergency departments, outpatient clinics, dialysis units, day-care units, or day-surgery settings were excluded according to the applicable ECDC protocol. The surveys included 276 patients in 2012 and 391 in 2022.

Trained local IPC personnel collected data using the standardized ECDC forms and definitions applicable in each survey year. Treating physicians assisted with verification when required. Sources included medical and nursing records, medication charts, and microbiology reports. Recorded variables included ward specialty, active HAI status, HAI site and origin, microbiological documentation, microorganisms and selected resistance markers, current antimicrobial prescriptions, indication, route, and surgical-prophylaxis duration.

### Protocol comparability

Both surveys used the ECDC PPS framework, but the 2012 and 2022 implementations differed in protocol version, available variables, HAI definitions and coding, and antimicrobial-susceptibility reporting.^9^ Protocol version 6.1 also included specific codes for healthcare-associated COVID-19, which had no equivalent in 2012. Bloodstream-infection and catheter-related-infection codes were present in version 4.3 output, but no such infections were recorded locally in 2012. The systemic-infection label also changed from “clinical sepsis” to “treated unidentified severe infection,” although the underlying definition was broadly retained. These factors, together with possible changes in diagnostic ascertainment and coding practice, limit direct comparison of infection-site distributions.

Patient-level reclassification of the 2012 observations under the 2022 protocol was impossible because only aggregate outputs were available. Overall patient-level HAI and AU prevalence were therefore regarded as the most comparable indicators. Infection-site distributions, microbiological documentation, antimicrobial indications, and susceptibility findings were treated as secondary descriptive outcomes. The principal protocol differences and their implications are summarized in Supplementary Table S1.

Susceptibility results were reported as protocol-year non-susceptibility markers. Under the earlier protocol, selected markers combined intermediate and resistant results in an IR/non-susceptible category. The 2022 survey recorded S, I, and R according to the revised EUCAST terminology, but the Greek aggregate analysis grouped results other than S as non-susceptible.^3,10,11^ Because testing practices and breakpoint versions could not be harmonized retrospectively, these percentages were not interpreted as resistance trends.

### Outcomes and statistical analysis

The primary outcome was patient-level prevalence of at least 1 active HAI. Secondary patient-level outcomes were receipt of at least 1 antimicrobial and specialty-specific HAI and AU prevalence. Categorical data are presented as counts and percentages. Fisher exact tests were used because several cells contained small numbers. Absolute percentage-point differences and prevalence ratios (PRs) with 95% confidence intervals (CIs), calculated using log-transformed standard errors, were reported for patient-level comparisons. No adjustment was made for multiple testing; specialty-specific P values were considered exploratory. A 2-sided *P* value less than .05 was used for the primary comparison.

The unit of analysis differed for other outcomes: the HAI case for infection sites and microbiological documentation, the isolated microorganism for organism distribution, the tested isolate for resistance markers, the antimicrobial agent for treatment indication, and the prophylaxis prescription for duration. A patient could contribute more than 1 HAI, microorganism, antimicrobial agent, or prophylaxis prescription. Because the aggregate outputs did not permit reconstruction of within patient clustering, these non-patient-level outcomes were summarized without inferential testing. Multivariable adjustment for age, comorbidity, device exposure, length of stay, illness severity, and other potential confounders was not possible. Analyses were performed with IBM SPSS Statistics, version 27 (IBM Corp, Armonk, New York).

### Ethical considerations

The original point-prevalence surveys were conducted with approval from the Institutional Review Board of University Hospital of Ioannina (Approval No. [13/14-6-2022(1)]). The present study involved a secondary analysis of anonymized aggregate data derived from these previously approved surveys, with no new data collection or contact with participants. No directly identifiable information was available to the investigators, and additional individual infor1med consent was therefore not required.

## Results

### Overall HAI and antimicrobial-use prevalence

At the 2012 survey, 23/276 patients (8.3%) had at least 1 HAI; at the 2022 survey, 48/391 (12.3%) had at least 1 HAI. The observed absolute difference was 3.9 percentage points (PR, 1.47; 95% CI, 0.92–2.36; *P* = .126). The 23 infected patients in 2012 contributed 23 HAI episodes. The 48 infected patients in 2022 contributed 58 HAI episodes (1.21 episodes per infected patient), including 3 episodes coded as healthcare-associated COVID-19. AU prevalence was 147/276 (53.3%) in 2012 and 206/391 (52.7%) in 2022 (PR, 0.99; 95% CI, 0.86–1.14; *P* = .937) (Table 1).

**Table 1.** Overall healthcare-associated infection and antimicrobial-use indicators, 2012 and 2022 Table 1 long description.

Indicator	2012	2022	Absolute difference	PR, 2022 vs 2012 (95% CI); P value
Surveyed patients	276	391	—	—
Patients with ≥1 HAI	23 (8.3%)	48 (12.3%)	+3.9 percentage points	1.47 (.92–2.36); .126
Total HAI episodes	23	58	—	—
HAI episodes per infected patient	1.00	1.21	—	—
Microbiologically documented HAI episodes	10/23 (43.5%)	38/58 (65.5%)	+22.0 percentage points	Descriptive
Patients receiving ≥1 antimicrobial	147 (53.3%)	206 (52.7%)	−.6 percentage points	.99 (.86–1.14); .937
Total antimicrobial agents	268	331	—	—
Antimicrobial agents per treated patient	1.82	1.61	—	—

*Note.* CI, confidence interval; HAI, healthcare-associated infection; PR, prevalence ratio. PRs and P values are reported only for independent patient-level outcomes. Other indicators use HAI episodes or antimicrobial agents as the unit of analysis and are descriptive.

### Clinical-specialty patterns

The ICU had the highest observed HAI prevalence in both surveys: 7/23 patients (30.4%) in 2012 and 6/21 (28.6%) in 2022. HAI prevalence was 6/111 (5.4%) and 13/128 (10.2%) in surgical specialties and 10/115 (8.7%) and 29/194 (14.9%) in medical specialties, respectively; the corresponding CIs were wide. AU prevalence in surgical specialties was 60/111 (54.1%) in 2012 and 86/128 (67.2%) in 2022 (PR, 1.24; 95% CI, 1.01–1.53; *P* = .046). This nominally significant secondary result was exploratory and was not adjusted for multiple comparisons or case mix. The comparable specialty groups contained all 276 patients in 2012 and 361/391 patients in 2022. The other 30 patients in 2022 were retained in hospital-level estimates but did not have a directly comparable 2012 specialty grouping (Table 2).

**Table 2.** Patient-level HAI and antimicrobial-use prevalence by clinical specialty Table 2 long description.

Outcome and specialty	2012, n/N (%)	2022, n/N (%)	PR (95% CI)	P value
**Panel A. Patients with ≥1 HAI**				
Surgical specialties	6/111 (5.4)	13/128 (10.2)	1.88 (.74–4.78)	.232
Medical specialties	10/115 (8.7)	29/194 (14.9)	1.72 (.87–3.40)	.155
Intensive care unit	7/23 (30.4)	6/21 (28.6)	.94 (.38–2.35)	1.000
Pediatrics/neonatology	0/27 (.0)	0/18 (.0)	Not estimable	1.000
Other specialty categories (2022 only)	Not applicable	0/30 (.0)	Not applicable	—
All specialties	23/276 (8.3)	48/391 (12.3)	1.47 (.92–2.36)	.126
**Panel B. Patients receiving ≥1 antimicrobial**				
Surgical specialties	60/111 (54.1)	86/128 (67.2)	1.24 (1.01–1.53)	.046
Medical specialties	65/115 (56.5)	97/194 (50.0)	.88 (.71–1.09)	.290
Intensive care unit	13/23 (56.5)	11/21 (52.4)	.93 (.54–1.59)	1.000
Pediatrics/neonatology	9/27 (33.3)	9/18 (50.0)	1.50 (.74–3.04)	.355
Other specialty categories (2022 only)	Not applicable	3/30 (10.0)	Not applicable	—
All specialties	147/276 (53.3)	206/391 (52.7)	.99 (.86–1.14)	.937

Note. AU, antimicrobial use; CI, confidence interval; HAI, healthcare-associated infection; PR, prevalence ratio. Fisher exact tests were used. Specialty comparisons were secondary and unadjusted; the nominal P value for surgical AU should be interpreted as exploratory. The exact labels for the 30 patients in 2022 without a comparable 2012 specialty grouping were unavailable in the aggregate output.

### HAI sites and microbiology

**Figure 1.**
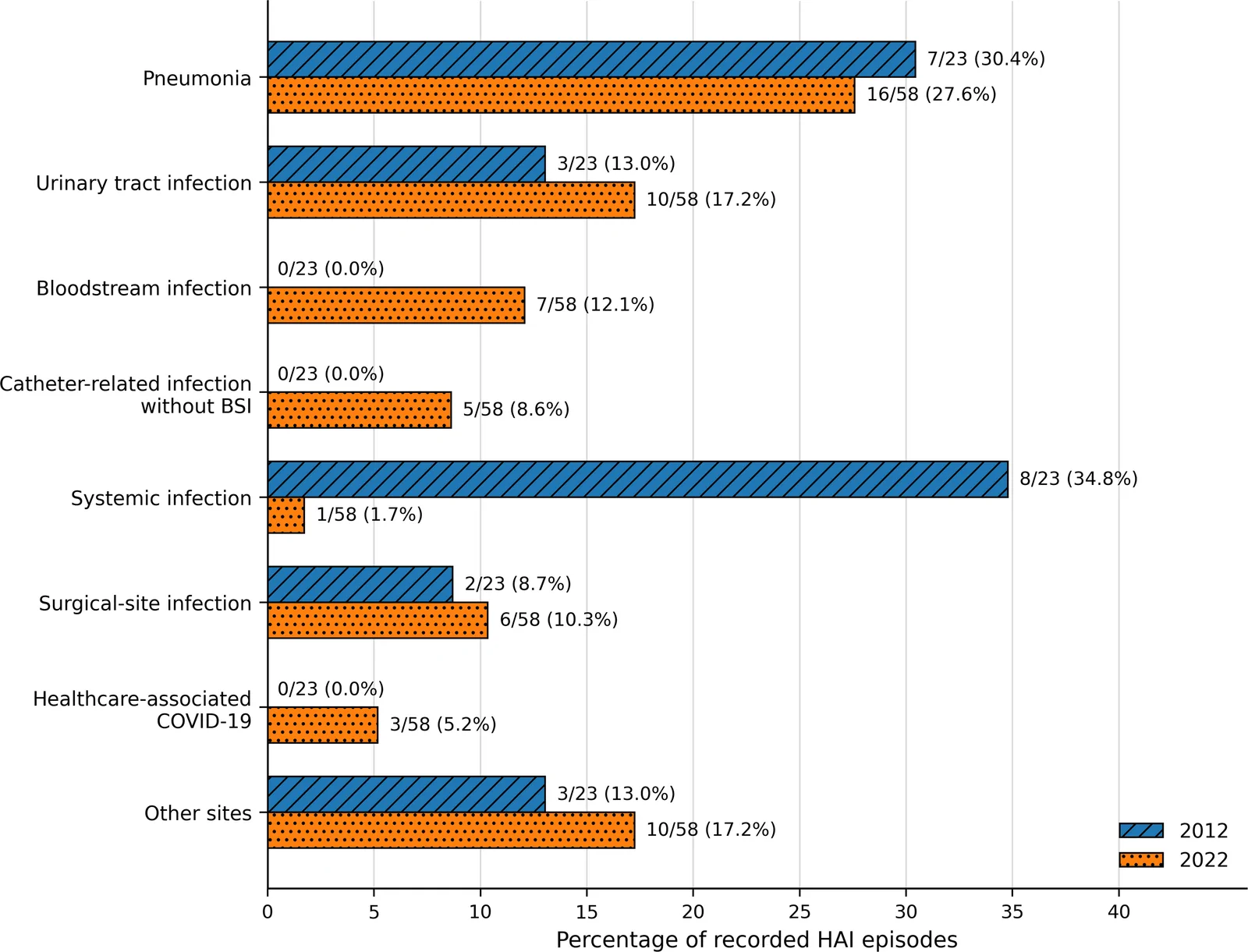
Distribution of recorded healthcare-associated infection sites in 2012 and 2022. Bar labels show n/N (%), where N is the total number of HAI episodes recorded in each survey (23 in 2012 and 58 in 2022). The 2022 denominator includes 3 healthcare-associated COVID-19 episodes. Other sites comprise lower respiratory tract infections other than pneumonia and infections of the central nervous system, cardiovascular system, eye/ear/nose/throat, gastrointestinal tract, and skin or soft tissue. Abbreviations: BSI, bloodstream infection; HAI, healthcare-associated infection.

Among all HAI episodes, pneumonia accounted for 7/23 (30.4%) in 2012 and 16/58 (27.6%) in 2022; urinary tract infections accounted for 3/23 (13.0%) and 10/58 (17.2%), respectively. In 2022, bloodstream infections accounted for 7/58 (12.1%), catheter-related infections without bloodstream infection for 5/58 (8.6%), and healthcare-associated COVID-19 for 3/58 (5.2%). No bloodstream, catheter-related, or COVID-19 infections were recorded in 2012. Systemic infections accounted for 8/23 (34.8%) in 2012 and 1/58 (1.7%) in 2022. These comparisons are descriptive because the surveys differed in protocol version, season, case mix, diagnostic ascertainment, and coding practices (Figure 1 and Supplementary Table S2).

Microbiological documentation was available for 10/23 HAI episodes (43.5%) in 2012 and 38/58 (65.5%) in 2022. Fifteen microorganisms were reported in 2012 and 44 in 2022. Protocol-year carbapenem non-susceptibility markers were reported for 4/5 A. baumannii, 1/5 P. aeruginosa, and 1/2 Klebsiella species isolates in 2012 and for 9/10, 4/4, and 4/5 tested isolates, respectively, in 2022. Because these results were based on small numbers and could not be reinterpreted using common breakpoints, they are presented only as local surveillance signals (Table 3 and Supplementary Tables S3–S4).

**Table 3.** Descriptive microbiological and protocol-specific non-susceptibility indicators, 2012 and 2022 Table 3 long description.

Indicator and unit of analysis	2012	2022
Microbiologically documented HAI cases (HAI case)	10/23 (43.5%)	38/58 (65.5%)
Total microorganisms isolated (microorganism)	15	44
*Acinetobacter baumannii* (microorganism)	5 (33.3%)	10 (22.7%)
*Klebsiella pneumoniae* (microorganism)	2 (13.3%)	7 (15.9%)
*Pseudomonas aeruginosa* (microorganism)	5 (33.3%)	4 (9.1%)
*A. baumannii* with recorded carbapenem non-susceptibility marker (tested isolate)	4/5 (80.0%)	9/10 (90.0%)
*P. aeruginosa* with recorded carbapenem non-susceptibility marker (tested isolate)	1/5 (20.0%)	4/4 (100.0%)
*Klebsiella* species with recorded carbapenem non-susceptibility marker (tested isolate)	1/2 (50.0%)	4/5 (80.0%)

Note. HAI, healthcare-associated infection. Values represent protocol-year non-susceptibility markers. Because susceptibility terminology, breakpoint versions, and testing practices could not be harmonized retrospectively, these percentages should not be interpreted as resistance trends. More than one isolate per patient may be represented.

### Antimicrobial indications and surgical prophylaxis

Treatment accounted for 169/268 antimicrobial agents (63.1%) in 2012 and 178/331 (53.8%) in 2022. Medical prophylaxis or chemoprophylaxis accounted for 36/268 (13.4%) and 76/331 (23.0%), whereas surgical prophylaxis accounted for 58/268 (21.6%) and 48/331 (14.5%), respectively. Among surgical-prophylaxis courses, 51/58 (87.9%) in 2012 and 47/48 (97.9%) in 2022 continued for more than 1 day (Table 4). These agent- and course-level findings are descriptive because patients could contribute more than 1 antimicrobial.

**Table 4.** Descriptive antimicrobial indications and surgical-prophylaxis duration Table 4 long description.

Indicator and unit of analysis	2012, n (%)	2022, n (%)	Absolute difference
Treatment (antimicrobial agent)	169 (63.1)	178 (53.8)	−9.3 percentage points
Community-acquired infection (antimicrobial agent)	101 (37.7)	97 (29.3)	−8.4 percentage points
Hospital/healthcare-associated infection (antimicrobial agent)	66 (24.6)	77 (23.3)	−1.4 percentage points
Long-term care-related infection (antimicrobial agent)	2 (.7)	4 (1.2)	+.5 percentage points
Surgical prophylaxis (antimicrobial agent)	58 (21.6)	48 (14.5)	−7.1 percentage points
Single-dose prophylaxis (prophylaxis prescription)	2/58 (3.4)	0/48 (.0)	−3.4 percentage points
One-day prophylaxis (prophylaxis prescription)	5/58 (8.6)	1/48 (2.1)	−6.5 percentage points
Prolonged prophylaxis >1 day (prophylaxis prescription)	51/58 (87.9)	47/48 (97.9)	+10.0 percentage points
Medical prophylaxis/chemoprophylaxis (antimicrobial agent)	36 (13.4)	76 (23.0)	+9.5 percentage points
Other indication (antimicrobial agent)	5 (1.9)	18 (5.4)	+3.6 percentage points
Unknown indication (antimicrobial agent)	0 (.0)	11 (3.3)	+3.3 percentage points

Note. Broad indication percentages use antimicrobial agents as the denominator (268 in 2012 and 331 in 2022). Duration percentages use surgical-prophylaxis prescriptions as the denominator (58 in 2012 and 48 in 2022). Patients could contribute more than 1 agent or prescription; findings are descriptive.

## Discussion

These two institutional PPSs provide cross-sectional snapshots of HAI and AU burden at two survey dates a decade apart, rather than a longitudinal assessment of change. Both surveys identified a substantial HAI burden, similar observed hospital-level AU prevalence, high HAI prevalence in the ICU, and very frequent continuation of surgical prophylaxis beyond 1 day. The observed difference in HAI prevalence from 8.3% to 12.3% was not statistically significant and had a wide CI; it should therefore be regarded as a surveillance signal rather than evidence of a continuous 10-year increase or institutional deterioration. Repeated surveillance in Catalonia showed that HAI and antibiotic-use trajectories can be non-linear and need not move in parallel.^12^ The 2022 estimate was close to the 12.1% reported nationally in Greece but higher than estimates from the 2022 German PPS and Lombardy.^3,13,14^ Within the 2022 Greek national PPS, HAI prevalence was 14.3% in tertiary hospitals, 7.5% in secondary hospitals, and 3.5% in pediatric hospitals; the corresponding AU prevalences were 64.9%, 53.5%, and 39.5%. These figures illustrate the influence of hospital type and provide descriptive context rather than risk-adjusted benchmarks.^3^ The wider ECDC PPS included 309,504 patients from 1,332 hospitals, but formal comparison with those hospitals was not possible using the available aggregate institutional data.^2^ The contribution of the present study is therefore the identification of institution-specific priorities rather than the estimation of a national or European trend.

The ICU had the highest HAI prevalence in both surveys, affecting 7/23 patients (30.4%) in 2012 and 6/21 (28.6%) in 2022. This pattern is consistent with regional PPS findings and with the established concentration of pneumonia and device-associated infection risk in critical care.^8,14,15^ Because device use, severity, prior antimicrobial exposure, and length of stay were unavailable, the present analysis cannot determine which factors explain the ICU prevalence or the descriptive differences in medical and surgical wards. The apparent rise in surgical AU deserves focused audit, but its nominal P value arose from an unadjusted secondary comparison among several specialty tests.

Pneumonia was the most frequently recorded HAI category at both survey dates, supporting continued emphasis on respiratory-infection prevention. By contrast, higher recorded proportions of urinary, bloodstream, and catheter-related infections and the sharp reduction in systemic infections cannot be interpreted as definitive temporal changes. Bloodstream- and catheter-related-infection categories were available under protocol version 4.3, but no such infections were recorded locally in 2012. Protocol revisions, improved microbiological ascertainment, changes in diagnostic and coding practices, and differences in patient case mix may have contributed. Prospective incidence surveillance with common definitions and device denominators would be required to distinguish true changes in acquisition risk from changes in detection and classification.

The different survey seasons further limit comparison. The 2012 survey was conducted during June–July, whereas the 2022 survey was conducted during November. Differences in respiratory-pathogen circulation, hospital activity, occupancy, and patient case mix could have influenced pneumonia and overall HAI prevalence. Because each year was represented by a single survey period, seasonal effects cannot be separated from other between-survey differences.

The interval between the surveys also encompassed the COVID-19 pandemic, which may have affected admission patterns, bed use, case mix, diagnostic testing, IPC practices, and antimicrobial prescribing. In addition, protocol version 6.1 included healthcare-associated COVID-19 categories that had no equivalent in 2012. No comparable intermediate PPS series was available, and the aggregate datasets did not contain standardized information on the timing, implementation, or effectiveness of intervening hospital policies or quality-improvement initiatives. Consequently, the study cannot determine whether the 2022 observations reflect pandemic-associated changes, subsequent stabilization, or the effect of a particular institutional intervention.

The higher proportion of microbiologically documented HAI episodes in 2022 may facilitate targeted therapy and local epidemiologic assessment, but it does not by itself prove improved diagnostic performance. Testing availability, specimen collection, infection-site distribution, and data completeness may also have changed. The non-susceptibility findings similarly require caution. High protocol-specific carbapenem non-susceptibility markers among *A. baumannii* and other Gram-negative isolates are plausible in the Greek tertiary-care context and are consistent with longitudinal surveillance from western Greece and broader ICU evidence.^16–18^ However, the denominators were small, patients may have contributed multiple isolates, and the 2 surveys used non-equivalent susceptibility classifications. A deduplicated, isolate-level time series using consistent breakpoints is needed before inferring a temporal change in antimicrobial resistance.

Similar AU prevalence at the two survey dates should not be equated with appropriate prescribing. More than half of patients received an antimicrobial in both surveys, and the overall patient-level prevalence concealed important differences in specialty, indication, and duration. The most actionable finding was prolonged surgical prophylaxis: 51/58 prescriptions (87.9%) in 2012 and 47/48 (97.9%) in 2022 exceeded 1 day. For comparison, 1,253/1,656 surgical-prophylaxis prescriptions (75.7%) exceeded 1 day in the 2022 Greek national PPS.^3^ Although this comparison was not adjusted for procedural case mix, the magnitude of prolonged use at both local survey dates identifies surgical prophylaxis as a high-priority stewardship target.

Current surgical-site-infection prevention guidance recommends discontinuation after incision closure for most procedures because continuation does not add preventive benefit and increases antimicrobial exposure.^19^ Nevertheless, the aggregate PPS data did not include procedure type, timing of the first dose, intraoperative redosing, allergy status, or documented exceptions. The appropriateness of each individual prescription could therefore not be assessed. A focused stewardship bundle should include procedure-specific guidance, documentation of indication and intended stop time, electronic or pharmacist-supported stop mechanisms, prospective audit and feedback, and department-level reporting. Audits should measure the proportion of prophylaxis prescriptions discontinued within 24 hours and report performance by surgical department and procedure type. A Greek tertiary-hospital program has shown that locally monitored stewardship can improve prescribing indicators,^20^ and systematic stewardship interventions can reduce resistant organisms and *Clostridioides difficile* infection.^21^

This study has strengths: both surveys were conducted at the same institution, covered eligible inpatients in participating wards, used the ECDC framework, and assessed complementary epidemiologic and stewardship indicators within the same institution. Its limitations are equally important. PPSs estimate prevalence rather than incidence and preferentially capture conditions associated with longer stays. The protocol versions differed; only aggregate outputs were available; case mix, device exposure, and illness severity could not be adjusted; non-patient outcomes could not account for clustering; multiple secondary comparisons were unadjusted; and isolate counts were small with nonharmonized susceptibility interpretation. The surveys were also conducted in different seasons and were separated by the COVID-19 pandemic. Furthermore, the available data did not permit evaluation of intervening IPC, AMS, or other quality-improvement policies. Because this was a single tertiary referral hospital with modest specialty and isolate counts, the findings should not be generalized to institutions with different case mix, referral roles, resources, or surveillance practices. The findings are therefore most useful for institutional priority setting, not causal inference. Repeated surveillance should now be linked to measurable interventions, particularly reduction of prophylaxis beyond 24 hours and prospective monitoring of respiratory and device-associated HAIs.

## Supporting information

10.1017/ash.2026.10912.sm001 Karavasili et al. supplementary material Karavasili et al. supplementary material

## Supplementary material

The supplementary material for this article can be found at https://doi.org/10.1017/ash.2026.10912.

## Research transparency and reproducibility

The aggregate institutional surveillance outputs are not publicly available but may be obtained from the corresponding author with institutional approval. Analyses were conducted in IBM SPSS Statistics; no analytic code was generated.

## Table 1. Long description

A table comparing healthcare-associated infection and antimicrobial-use indicators between 2012 and 2022. The table has 10 rows and 5 columns. Column headers are Indicator, 2012, 2022, Absolute difference, and PR, 2022 vs 2012 (95% CI); P value. Row labels include Surveyed patients, Patients with ≥1 HAI, Total HAI episodes, HAI episodes per infected patient, Microbiologically documented HAI episodes, Patients receiving ≥1 antimicrobial, Total antimicrobial agents, and Antimicrobial agents per treated patient. Notable trends include an increase in patients with ≥1 HAI from 23 in 2012 to 48 in 2022, and an increase in total HAI episodes from 23 in 2012 to 58 in 2022. The prevalence of antimicrobial use slightly decreased from 53.3% in 2012 to 52.7% in 2022.

Navigate back to Table 1..

## Table 2. Long description

A table comparing patient-level HAI and antimicrobial-use prevalence by clinical specialty across years 2012 and 2022. The table has two panels: Panel A and Panel B. Panel A focuses on patients with at least one healthcare-associated infection (HAI), while Panel B focuses on patients receiving at least one antimicrobial. Each panel lists different specialties and provides data for the years 2012 and 2022, including the number of patients with HAI or receiving antimicrobials, the prevalence ratio (PR), confidence intervals (CI), and P values. Panel A includes rows for surgical specialties, medical specialties, intensive care unit, pediatrics/neonatology, and other specialty categories. Panel B includes similar rows but focuses on antimicrobial use. Notable trends include the highest observed HAI prevalence in the ICU for both years and an increase in antimicrobial use in surgical specialties from 2012 to 2022.

Navigate back to Table 2..

## Table 3. Long description

A table comparing microbiological indicators and non-susceptibility markers for the years 2012 and 2022. The table has 8 rows and 3 columns. Column headers are Indicator and unit of analysis, 2012, and 2022. Row 1: Microbiologically documented HAI cases (HAI case), 10/23 (43.5 percent), 38/58 (65.5 percent). Row 2: Total microorganisms isolated (microorganism), 15, 44. Row 3: Acinetobacter baumannii (microorganism), 5 (33.3 percent), 10 (22.7 percent). Row 4: Klebsiella pneumoniae (microorganism), 2 (13.3 percent), 7 (15.9 percent). Row 5: Pseudomonas aeruginosa (microorganism), 5 (33.3 percent), 4 (9.1 percent). Row 6: A. baumannii with recorded carbapenem non-susceptibility marker (tested isolate), 4/5 (80.0 percent), 9/10 (90.0 percent). Row 7: P. aeruginosa with recorded carbapenem non-susceptibility marker (tested isolate), 1/5 (20.0 percent), 4/4 (100.0 percent). Row 8: Klebsiella species with recorded carbapenem non-susceptibility marker (tested isolate), 1/2 (50.0 percent), 4/5 (80.0 percent).

Navigate back to Table 3..

## Table 4. Long description

The table has 11 rows and 4 columns. The columns are labeled Indicator and unit of analysis, 2012, n (%), 2022, n (%), and Absolute difference. The rows list different indicators such as Treatment (antimicrobial agent), Community-acquired infection (antimicrobial agent), Hospital/healthcare-associated infection (antimicrobial agent), Long-term care-related infection (antimicrobial agent), Surgical prophylaxis (antimicrobial agent), Single-dose prophylaxis (prophylaxis prescription), One-day prophylaxis (prophylaxis prescription), Prolonged prophylaxis>1 day (prophylaxis prescription), Medical prophylaxis/chemoprophylaxis (antimicrobial agent), Other indication (antimicrobial agent), and Unknown indication (antimicrobial agent). Each row provides the percentage and count of antimicrobial agents for the years 2012 and 2022, along with the absolute difference between these years. Notable trends include a decrease in the percentage of treatments and surgical prophylaxis and an increase in medical prophylaxis/chemoprophylaxis and unknown indications.

Navigate back to Table 4..
