# Supplementary material for: Healthcare-associated infections and antimicrobial use in a Greek tertiary hospital: two cross-sectional point-prevalence surveys in 2012 and 2022
Source: Antimicrob Steward Healthc Epidemiol. 2026 Oct 8;6(1):e328. doi: 10.1017/ash.2026.10912 (PMC13760297; doi:10.1017/ash.2026.10912)
Supplement: Supplementary file 1 [file S2732494X26109127sup001.docx]

Supplementary Table S1. Principal differences between the 2012 and 2022 survey protocols and implications for comparability

| **Domain** | **2012: ECDC v4.3** | **2022: ECDC v6.1 framework** | **Interpretive consequence** |
| --- | --- | --- | --- |
| Survey implementation | Patient-based standard implementation | Greek national unit-based/light implementation | Overall eligible-patient denominators remain usable; comparable individual covariates were unavailable. |
| Eligible population | Inpatients admitted before 08:00, with protocol-specified exclusions | Same broad eligibility rule | Overall HAI and AU prevalence are the most comparable endpoints. |
| Active HAI definition | Protocol-year active HAI definitions | Updated protocol-year definitions | Retrospective reclassification was impossible; prevalence comparison is unadjusted. |
| COVID-19 | No specific HAI category | COV-ASY, COV-MM, and COV-SEV categories available | COVID-19 episodes have no 2012 counterpart and should be identified separately. |
| Infection-site coding | PN, UTI, SSI, BSI, CRI, SYS, and other categories available | Same broad categories, with updates to definitions and coding | Site distributions are descriptive; BSI and CRI had zero local counts in 2012. |
| Systemic infection | SYS-CSEP described as clinical sepsis | Renamed treated unidentified severe infection; underlying definition broadly retained | The large distributional change may reflect coding or ascertainment and is not evidence of a true trend. |
| Antimicrobial use | Patient exposure, indication, and prophylaxis duration recorded | Same broad concepts, with updated coding/ATC framework | Overall AU and prolonged-prophylaxis measures are cautiously comparable; detailed indication distributions are descriptive. |
| Susceptibility | Protocol markers using S versus IR/non-susceptible coding | S/I/R recorded under revised EUCAST terminology; Greek reporting grouped non-S results as NS | Report as protocol-year non-susceptibility; do not interpret as a harmonized resistance trend. |
| Available analytical data | Aggregate hospital output | Aggregate hospital output | No patient-level reclassification, case-mix adjustment, deduplication, or clustering correction was possible. |

Supplementary Table S2. Detailed distribution of healthcare-associated infection sites

| **HAI site** | **2012 n (%) of HAIs** | **2022 n (%) of HAIs** | **Absolute difference** |
| --- | --- | --- | --- |
| Pneumonia | 7 (30.4%) | 16 (27.6%) | -1.3 pp |
| Other lower respiratory tract infections | 2 (8.7%) | 3 (5.2%) | -3.2 pp |
| Surgical site infections | 2 (8.7%) | 6 (10.3%) | +2.2 pp |
| Urinary tract infections | 3 (13.0%) | 10 (17.2%) | +5.1 pp |
| Bloodstream infections | 0 (0.0%) | 7 (12.1%) | +12.7 pp |
| Catheter-related infections without BSI | 0 (0.0%) | 5 (8.6%) | +9.1 pp |
| Gastrointestinal infections | 1 (4.3%) | 1 (1.7%) | -2.5 pp |
| Skin and soft tissue infections | 0 (0.0%) | 3 (5.2%) | +5.5 pp |
| Systemic infections | 8 (34.8%) | 1 (1.7%) | -33.0 pp |
| Other sites | 0 (0.0%) | 3 (5.2%) | +5.5 pp |

Note. The unit of analysis was the HAI episode. Percentages are based on 23 HAI episodes in 2012 and 58 HAI episodes in 2022. Individual patients could contribute more than one HAI episode. Because within-patient clustering could not be reconstructed from the aggregate PPS outputs, all comparisons are descriptive and no inferential P values are reported. BSI, bloodstream infection; pp, percentage points.

Supplementary Table S3. Descriptive distribution of microorganisms isolated from HAI episodes

| **Microbiological indicator** | **2012** | **2022** |
| --- | --- | --- |
| HAI episodes with microbiological documentation | 10/23 (43.5%) | 38/58 (65.5%) |
| Total microorganisms isolated | 15 | 44 |
| Gram-negative non-Enterobacteriaceae | 10 (66.7%) | 14 (31.8%) |
| Acinetobacter baumannii | 5 (33.3%) | 10 (22.7%) |
| Pseudomonas aeruginosa | 5 (33.3%) | 4 (9.1%) |
| Enterobacteriaceae | 5 (33.3%) | 15 (34.1%) |
| Klebsiella spp. / K. pneumoniae | 2 (13.3%) | 7 (15.9%) |
| Escherichia coli | 2 (13.3%) | 4 (9.1%) |
| Enterococcus spp. | 0 (0.0%) | 6 (13.6%) |
| Staphylococcus aureus | 0 (0.0%) | 2 (4.5%) |
| Candida spp. | 0 (0.0%) | 2 (4.5%) |
| SARS-CoV-2 | 0 (0.0%) | 3 (6.8%) |

Note. Microbiological-documentation percentages are based on HAI episodes, whereas microorganism percentages are based on all isolated microorganisms: 15 in 2012 and 44 in 2022. Individual patients could contribute more than one HAI episode or microorganism. Because within-patient clustering could not be reconstructed, the findings are descriptive and no inferential P values are reported.

Supplementary Table S4. Descriptive antimicrobial-resistance markers among tested isolates

| **Resistance marker** | **2012** | **2022** |
| --- | --- | --- |
| MRSA among Staphylococcus aureus | 0/0 (NA) | 2/2 (100.0%) |
| VRE among Enterococcus spp. | 0/0 (NA) | 5/5 (100.0%) |
| Enterobacteriaceae with recorded 3GC resistance marke | 5/5 (100.0%) | 5/12 (41.7%) |
| E. coli with recorded 3GC resistance marker | 2/2 (100.0%) | 1/3 (33.3%) |
| Klebsiella spp. with recorded 3GC resistance marker | 2/2 (100.0%) | 4/7 (57.1%) |
| Enterobacteriaceae with recorded carbapenem resistance marker | 3/5 (60.0%) | 4/9 (44.4%) |
| Klebsiella spp. with recorded carbapenem resistance marker | 1/2 (50.0%) | 4/5 (80.0%) |
| *P.* aeruginosa with recorded carbapenem resistance marker | 1/5 (20.0%) | 4/4 (100.0%) |
| A. baumannii with recorded carbapenem resistance marker | 4/5 (80.0%) | 9/10 (90.0%) |

Note. The unit of analysis was the tested isolate. Denominators represent isolates with an available result for the relevant resistance marker and may therefore be smaller than the total number of isolates of that microorganism. Individual patients could contribute more than one isolate. Findings are descriptive because within-patient clustering could not be addressed, denominators were small, and resistance classifications were not directly harmonized between the two protocol periods.
